# Multiple Losses of MSH1, Gain of mtMutS, and Other Changes in the MutS Family of DNA Repair Proteins in Animals

**DOI:** 10.1093/gbe/evab191

**Published:** 2021-08-17

**Authors:** Viraj Muthye, Dennis V Lavrov

**Affiliations:** Department of Ecology, Evolution and Organismal Biology, Iowa State University, Ames, Iowa

**Keywords:** mitochondria, MutS, DNA repair, octocorals, cnidarians

## Abstract

MutS is a key component of the mismatch repair (MMR) pathway. Members of the MutS protein family are present in prokaryotes, eukaryotes, and viruses. Six MutS homologs (MSH1–6) have been identified in yeast, of which three function in nuclear MMR, while MSH1 functions in mitochondrial DNA repair. MSH proteins are believed to be well conserved in animals, except for MSH1—which is thought to be lost. Two intriguing exceptions to this general picture have been found, both in the class Anthozoa within the phylum Cnidaria. First, an ortholog of the yeast-MSH1 was reported in one hexacoral species. Second, a MutS homolog (mtMutS) has been found in the mitochondrial genome of all octocorals. To understand the origin and potential functional implications of these exceptions, we investigated the evolution of the MutS family both in Cnidaria and in animals in general. Our study confirmed the acquisition of octocoral mtMutS by horizontal gene transfer from a giant virus. Surprisingly, we identified MSH1 in all hexacorals and several sponges and placozoans. By contrast, MSH1 orthologs were lacking in other cnidarians, ctenophores, and bilaterian animals. Furthermore, while we identified MSH2 and MSH6 in nearly all animals, MSH4, MSH5, and, especially, MSH3 were missing in multiple species. Overall, our analysis revealed a dynamic evolution of the MutS family in animals, with multiple losses of MSH1, MSH3, some losses of MSH4 and MSH5, and a gain of the octocoral mtMutS. We propose that octocoral mtMutS functionally replaced MSH1 that was present in the common ancestor of Anthozoa.

Significance

Animal mitochondrial genomes (mtDNA) display some of the highest rates of sequence evolution, an observation previously explained by deficiencies in their mtDNA repair machinery. It is believed that animal mitochondria lack the mismatch repair (MMR) system. Here, we investigate the evolutionary history of MutS proteins—key players in MMR. We show that a mitochondria-targeted MutS homolog (MSH1) was likely present in the common ancestor of animals but has been lost in multiple lineages, including bilaterian animals. Surprisingly, a MutS homolog (mtMutS) was gained by octocoral cnidarian mitochondria, probably via horizontal gene transfer from a giant virus. Remarkably, lineages encoding either MSH1 or mtMutS have lower rates of mitochondrial sequence evolution than lineages without them.

## Introduction

DNA mismatch repair (MMR) is an evolutionary-conserved DNA repair pathway that plays an important role in maintaining genomic stability (Chatterjee and Walker 2017). MMR primarily corrects DNA mismatches generated during DNA replication that have escaped proof-reading by DNA polymerases and improves the fidelity of DNA replication by 50–1,000-fold (Hsieh and Yamane 2008; Hsieh and Zhang 2017). The deactivation of this pathway in mammalian cells has been associated with predisposition to various types of cancers (Papadopoulos et al. 1994), defects in meiosis (de Vries et al. 1999), and sterility (Baker et al. 1996). Defects in MMR also lead to an increase in the spontaneous mutation rate in bacteria (Tiraby and Fox 1973) and an accelerated rate of sequence evolution, longer microsatellites, and decreased GC content in fungi (Phillips et al. 2021).

The key proteins of MMR—MutS and MutL—are well-conserved in prokaryotes and eukaryotes (Eisen 1998). In *Escherichia coli*, a MutS homodimer recognizes and binds DNA mismatches. MutL interacts with MutS and recruits and activates the endonuclease MutH. MutH introduces a nick in the newly synthesized DNA strand that contains the error, which is followed by the excision and resynthesis of the error-containing strand (Liu et al. 2017). The key steps of MMR in *E. coli—*DNA mismatch identification by MutS proteins, error-strand discrimination, excision, and DNA resynthesis—are conserved in eukaryotes (Liu et al. 2017). However, there are two key differences: the underlying MMR machinery is more complex in eukaryotes and there are at least two genomes in most eukaryotic cells (nuclear and organellar) that replicate independent of each other and possibly require their own DNA repair pathways.

At least nine MutS subfamilies have been identified across eukaryotes, with MSH2–6 present in most species. Among these proteins, MSH2, MSH3, and MSH6 are involved in MMR in the nuclear genome, whereas MSH4 and MSH5 function in meiotic recombination (Eisen 1998). Unlike a single MutS homodimer in prokaryotes, two MutS heterodimers usually function in DNA mismatch recognition in eukaryotes—MutS*α* (MSH2 and MSH6) and MutS*β* (MSH2 and MSH3) (Fukui 2010; Liu et al. 2017). In mammals, MutS*α* binds to single-base mismatches and smaller (up to 3 nucleotides) indels (Gupta et al. 2011; Romanova and Crouse 2013), while MutS*β* recognizes longer (up to 13 nucleotides) indels (Fukui 2010; Liu et al. 2017).

While the function of MutS homologs in the nucleus has been well characterized, their involvement in mitochondrial DNA repair is less understood. Studies in plants and fungi have identified two MutS homologs targeted to the mitochondria: yeast-MSH1 and plant-MSH1 (Chi and Kolodner 1994; Abdelnoor et al. 2006). Although both have been named “MSH1”, plant-MSH1 and yeast-MSH1 are not orthologous. While yeast-MSH1 clusters with MSH2–6, the closest relatives of plant-MSH1 are found among giant viruses (Wu et al. 2020). Both yeast-MSH1 and plant-MSH1 are known to localize to mitochondria and function in DNA repair (Reenan and Kolodner 1992; Kaniak et al. 2009; Pogorzala et al. 2009; Wu et al. 2020). However, their involvement in MMR has not been demonstrated. While yeast-MSH1 has been shown to bind to mismatches (Chi and Kolodner 1994), it appears to function in base excision repair and homologous recombination instead of MMR (summarized in Chalissery et al. 2017). Furthermore, while some experimental analyses have reported MMR activity in mammalian mitochondria, no MutS proteins appear to be involved in it (Mason et al. 2003; de Souza-Pinto et al. 2009). In fact, no MutS homologs are known to localize to animal mitochondria, except for two remarkable exceptions within a single animal phylum: Cnidaria. First, an ortholog of the mitochondria-targeted yeast-MSH1 has been reported in the starlet sea anemone *Nematostella vectensis* (Bilewitch and Degnan 2011). Second, a gene for a MutS-like protein (mtMutS) has been found in mitochondrial genomes of octocoral cnidarians (Beagley et al. 1995; Beaton et al. 1998). While earlier studies assumed that the gene encoding octocoral mtMutS originated in the mitochondrial genome by transfer from the nuclear genome, more recent studies suggested a horizontal gene transfer (HGT) event, possibly from a giant virus (Ogata et al. 2011; Bilewitch and Degnan 2011). Interestingly, mtMutS possesses an “HNH” endonuclease domain at its C-terminus—a domain that is also found in MutS proteins from giant viruses (Bilewitch and Degnan 2011).

The presence of two instances of putative mitochondrial MutS homologs in one group of animals raises questions about the origin and evolution of the two MutS proteins. However, our understanding of these questions has been hampered by the lack of information on the composition and evolution of the whole MutS family in Cnidaria. The rapid increase in the availability of genomic data removed this obstacle and allowed us to characterize the composition of MutS homologs in all major cnidarian groups—Anthozoa, Medusozoa, and Endocnidozoa. In addition, we compared the evolution of the MutS family in Cnidaria with that among all major animal phyla.

## Results

### Characterization of MutS Homologs in Animals

**Fig. 1.**
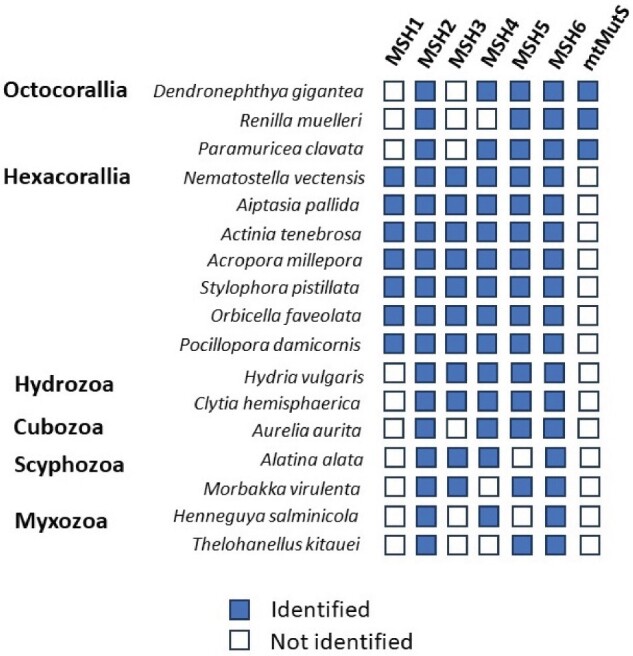
The distribution of MutS homologs in phylum Cnidaria. MSH1–6 are encoded in the nuclear genome and mtMutS is encoded in the mitochondrial genome.

**Fig. 2.**
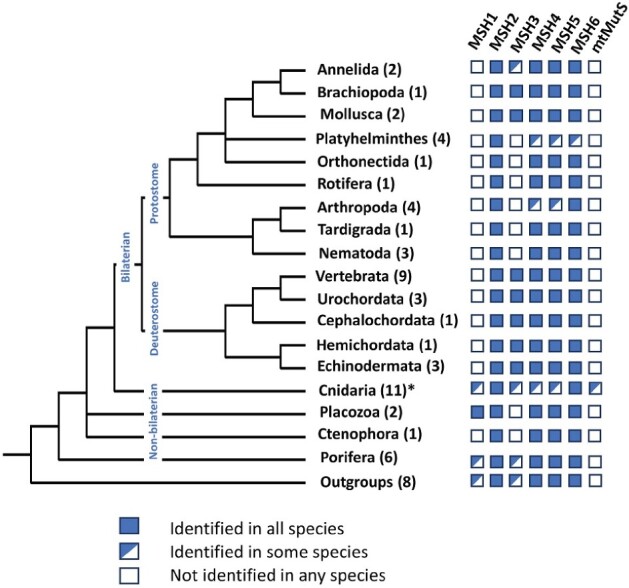
The distribution of MutS homologs across animal phyla. The numbers in parentheses next to the taxonomic groups denote the number of species from each group included in the analysis. MSH1–6 are encoded in the nuclear genome and mtMutS is encoded in the mitochondrial genome. Only phylum Cnidaria has the mitochondria-encoded mtMutS protein (marked with an *). The phylogenetic tree used in this image was taken from (Guijarro-Clarke et al. 2020).

To understand the origin and putative function of the octocoral mtMutS protein, we investigated the presence/absence of nuclear-encoded MutS homologs in 17 published cnidarian genomes. MSH2 and MSH6 were identified in all of them, even in myxozoan genomes that are known to have experienced major gene-losses and elevated rates of sequence evolution (Holzer et al. 2018). By contrast, the distribution of the other MSH proteins varied across species (fig. 1). MSH3 was identified in 11/17 analyzed genomes, while MSH4 and MSH5 were found in 14/17 and 15/17, respectively. By extending our analysis to 57 animal and 8 outgroup species, we found a similar pattern of conservation in the MutS family. While MSH2 was identified in all analyzed genomes and MSH6 in all but one, other MutS homologs were lacking in multiple species. In particular, MSH3 was not found in the majority of ecdysozoan and lophotrochozoan species used for this study (fig. 2).

### Presence of MSH1 in Several Animal Lineages

Unexpectedly, we identified orthologs of fungal MSH1 in all hexacoral, two sponge (*Amphimedon queenslandica and Ephydatia muelleri*), as well as both placozoan (*Trichoplax adhaerens and Hoilungia hongkongensis*) genomes used for the study. In each of the placozoan species, several MSH1 copies were found (two in *T. adhaerens* and three in *H. hongkongensis*) that appear to represent Placozoa-specific duplications. MSH1 was also found in multiple outgroups, including choanoflagellates (*Monosiga brevicollis and Salpingoeca rosetta*), the closest relatives of animals (Lang et al. 2002), as well as *Capsaspora owczarzaki and Dictyostelium discoideum*. By contrast, no MSH1 orthologs were detected in the genomes of octocorals, medusozoan cnidarians, ctenophores, or bilaterian animals.

We analyzed several key features of the MSH1 proteins identified in this study: length, protein domain content, sequence identity to yeast-MSH1, and the presence of an N-terminus mitochondria-targeting signal (MTS). The length of MSH1 ranged from 609 amino acids in the pale anemone *Exaiptasia diaphana* (previously named *Aiptasia pallida*) to 1,014 amino acids in the starlet sea anemone *Nematostella vectensis*. The amino acid sequence identity of animal MSH1 to yeast-MSH1 ranged from 18% in the mountainous star coral *Orbicella faveolata* to 25% in the Waratah anemone *Actinia tenebrosa*. All complete MSH1 proteins contained the “MutS I” protein domain, known to function in DNA mismatch identification. A conserved phenylalanine residue (F77 in *N. vectensis*) critical for this function (Lamers et al. 2000; Obmolova et al. 2000) was found in all animal MSH1 proteins except the duplicated proteins in *T. adhaerens* and in *H. hongkongensis*, which contained an alanine at that position. Similarly, all animal MSH1 proteins contained the “MutS III” and “MutS V” domains present in yeast-MSH1. However, the “MutS II” was not identified in *Orbicella faveolata* (contributing to the lowest sequence identity with yeast-MSH1) and one of the MSH1 proteins in the placozoan *T. adhaerens*. An additional “MutS IV” domain, not found in yeast-MSH1, was identified in ten animal MSH1 proteins.

Finally, while yeast-MSH1 contains an N-terminus MTS that targets the protein to the mitochondria, such a sequence was found in only MSH1 proteins from 5 nonbilaterian species and 3 outgroup species. In particular, a canonical MTS was found in only three hexacoral species (supplementary folder S5, Supplementary Material online).

### Acquisition of mtMutS and Loss of MSH1 in Octocorals

While none of the octocoral genomes contained orthologs of yeast MSH1, all publicly available octocoral mitochondrial genomes encode mtMutS. The analyzed mtMutS proteins were well-conserved with respect to size and protein domain content (supplementary file S3, Supplementary Material online). The size of octocoral mtMutS ranged from 957 amino acids in *Paragorgia* sp. 1075761 to 1,022 amino acids in *Briareum asbestinum* (mean = 987 amino acids) (supplementary fig. S2, Supplementary Material online). The protein domain content of mtMutS was also well-conserved. All mtMutS proteins contained four domains: “MutS I,” “MutS III,” “MutS V,” and “HNH.” The mtMutS proteins from two species, *Briareum asbestinum and Sarcophyton trocheliophorum*, were predicted to contain a “MutS IV” domain, nested within the large “MutS III” domain. The “MutS II” protein domain was not found in any mtMutS protein.

**Fig. 3.**
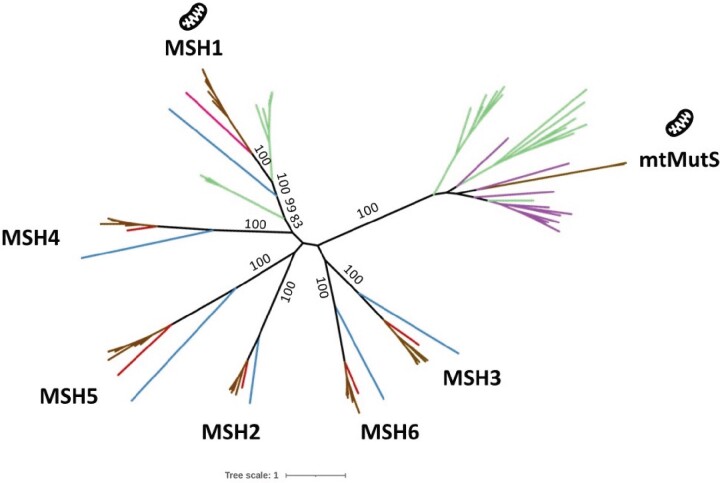
Maximum Likelihood tree of MutS homologs from cnidarians, bacteria, archaea, and viruses. Complete protein sequences were used to build this phylogeny using RAxML (1,000 boostraps). The branches are colored as follows: Cnidaria (brown), yeast (blue), human (red), *Trichoplax adhaerens* (pink), prokaryotes (green), and viruses (purple).

The additional cnidarian MSH sequences identified in this study allowed us to analyze phylogenetic relationships between cnidarian mtMutS and MSH1–6 proteins. To better understand the origin of mtMutS, we built a phylogeny with MSH homologs, mtMutS, and the ten best BLAST hits to mtMutS and MSH1 (fig. 3). The reconstructed phylogenetic tree showed that mtMutS is not related to MSH1 and is separated from all MSH subunits by a long branch. We identified a clade containing octocoral mtMutS and the MutS homologs from viruses and *ϵ*-proteobacteria. This clade corresponds to the clade labeled as “MutS7” in previous analyses (Bilewitch and Degnan 2011; Ogata et al. 2011). We also found that there were some bacterial sequences closely related to MSH1. The clade comprising MSH1 and the bacterial MutS sequences corresponds to the clade labeled as “MSH1/MutS1” in previous analyses (Bilewitch and Degnan 2011; Ogata et al. 2011). Hence, we searched for closest hits to all MSH homologs and found that most of them have closely related prokaryotic sequences (supplementary fig. S4, Supplementary Material online).

## Discussion

### The Evolution of the MSH Family in Animals

It is customary to say that MSH proteins are conserved across eukaryotes. Our study contradicts this notion and shows a spotty distribution of MSH3, MSH4, and MSH5 in animals. In particular, we were not able to identify MSH3—which, together with MSH2, forms the MutS*β* heterodimer—in several well-studied ecdysozoan and lophotrochozoan genomes. This result supports the previous reports of MSH3 losses in insects (Lin et al. 2007), including *Drosophila melanogaster* (Sekelsky et al. 2000; Sekelsky 2017), as well as the nematode *Caenorhabditis elegans* (Marti et al. 2002). When both MutS*α* and MutS*β* heterodimers are present, the loss of MutS*β* causes a weaker mutator phenotype compared to the loss of MutS*α* (Romanova and Crouse 2013). The remaining two MSH homologs with inferred losses in animals, MSH4 and MSH5, have roles in meiotic recombination instead of DNA repair (Eisen 1998). These two proteins are also known to be absent in the genome of *D. melanogaster*, where it has been proposed that other proteins have adopted their roles (Sekelsky et al. 2000).

There is an additional complication in our understanding of MutS family evolution in eukaryotes. While some studies suggest that MSH proteins form a monophyletic clade and that their evolution can be explained by several rounds of duplication of an ancestral MutS homolog, most phylogenetic studies reconstruct eukaryotic MSH family as a paraphyletic group with some bacterial sequences closely related to MSH1 (Bilewitch and Degnan 2011; Ogata et al. 2011) and other MSH proteins (supplementary fig. S4, Supplementary Material online). Further studies are required to clarify whether this result indicates HGT of eukaryotic *msh* gene to bacteria, multiple independent origins of eukaryotic MutS homologs, or contamination in the dataset.

### Orthologs of Yeast MSH1 in Nonbilaterian Animals

One of the surprising results of this study was the discovery of MSH1 orthologs in several phyla of nonbilaterian animals, as well as choanoflagellates. Although MSH1 was one of the first two MutS homologs found in eukaryotes, it has been identified in only one animal—the hexacoral *Nematostella vectensis* (Bilewitch and Degnan 2011). Our study showed that MSH1 is not restricted to hexacorals, and is present in at least two more nonbilateria phyla. Thus, it is likely that MSH1 was present in the common ancestor of all animals and lost multiple times. Because yeast-MSH1 is involved in mitochondrial DNA repair, we propose that a similar function of MSH1 was present early in animal evolution, and is still retained by some nonbilaterian animals. Interestingly, while yeast-MSH1 and the majority of MSH1 orthologs analyzed in this study were predicted to possess a MTS, majority of the hexacoral MSH1 proteins lacked a canonical MTS. However, this result does not preclude its mitochondrial localization. Our previous analysis has shown that several well-known mitochondrial proteins lacked canonical MTS in nonbilaterian taxa (Muthye and Lavrov 2018). Consistent with this observation, MSH1 is predicted to be imported to mitochondria by DeepMito and likely uses an alternative targeting signal for mitochondrial import. In our analyses, we found strong support for a clade comprising MSH1 and several eubacterial MutS proteins. However, we were unable to resolve relationships inside this clade owing to poor bootstrap support values.

### The HGT Origin of Octocoral mtMutS

The identification of MSH1 in hexacorals and MSH2–6 from all major cnidarian groups allowed us to re-examine the phylogenetic relationships among the MutS homologs in Cnidaria. Our results provide further support for the hypothesis that mtMutS was acquired via HGT from either a bacterium or a giant virus (Bilewitch and Degnan 2011; Ogata et al. 2011). The idea that giant viruses acted as vectors of mtMutS into octocoral mitochondria does have some additional support. First, HGT from giant viruses has likely happened one more time in the MutS family of proteins, with the transfer of plant-MSH1 in land plants (Wu et al. 2020). Second, giant viruses are among the main groups of viruses found on the octocoral *Gorgonia ventalina* (Hewson et al. 2012; Gudenkauf and Hewson 2016; van de Water et al. 2018) and MutS orthologs from giant viruses are abundant in marine environments (Ogata et al. 2011). Finally, viral proteins have replaced mitochondrial proteins of eubacterial origin before, including RNA polymerase, DNA polymerase, and the primase-helicase TWINKLE (Shutt and Gray 2006). However, mtMutS would be the first such HGT into the animal mitochondrial genome.

### MSh1, mtMutS, and Mitochondrial MMR in Animals

Although we do not know whether hexacoral MSH1 and octocoral mtMutS are functionally similar, several lines of evidence support this hypothesis. Based on its orthology to yeast-MSH1, conserved protein domain architectures, and the inferred mitochondrial localization, animal MSH1 are likely to be involved in mitochondrial DNA repair. Similarly, octocoral mtMutS has conserved protein domain content, and functional residues suggesting a conserved function (Bilewitch and Degnan 2011). The distribution of MSH1 and mtMutS in animals also provides insight into the function of the two proteins. Anthozoans display some of the lowest rates of mitochondrial sequence evolution in animals (Lavrov and Pett 2016) and also have either mtDNA-encoded (octocorals) or mitochondria-targeted MutS homologs (hexacorals). Higher rates of mitochondrial sequence evolution are observed in medusozoan and endocnidozoan cnidarians, as well as ctenophores and bilaterian animals, which lack mitochondrial MutS homologs. Similarly, in the phylum Porifera (sponges), MSH1 was identified in demosponges (low rate of mitochondrial sequence evolution), but not in calcarean sponges (higher rate of mitochondrial sequence evolution).

It is interesting to note that, unlike MSH1, mtMutS has an endonuclease domain, which suggests a potential self-contained MMR function. By contrast, if MSH1 functions in MMR, it would likely depend on additional molecular factors like MutL homologs for its activity. Additional research is being conducted by us and collaborators to characterize the function and structure of hexacoral MSH1 and octocoral mtMutS.

In conclusion, the results of this study suggest that a viral *mutS* gene has integrated into the mitochondrial genome of octocorals via HGT and functionally replaced the *msh1* gene that was present in the common ancestor of Anthozoa. Furthermore, we demonstrate that the *msh1* gene is present in several phyla of nonbilaterian animals and that its loss correlates with higher rates of sequence evolution in the mitochondrial genomes of ctenophores and bilaterian animals.

## Materials and Methods

### Data Acquisition

Predicted proteomes of *Homo sapiens* (human), *Mus musculus* (mouse), *Saccharomyces cerevisiae* (yeast), and *Arabidopsis thaliana* (thale-cress) were downloaded from UniProt (UniProt Consortium 2015). These species are referred to as “reference species” because their MSH proteins have been well-characterized. To characterize MSH proteins in the phylum Cnidaria, we downloaded protein models from the genomes of 17 cnidarian species (10 anthozoan, 5 medusozoan, and 2 endocnidozoan species) (supplementary file S1, Supplementary Material online). To identify MSH proteins across all major animal phyla, predicted proteins from the genomes of 57 animal species along with 8 nonanimal outgroup species were downloaded from sources listed in supplementary file S2, Supplementary Material online. For the analysis of the mtMutS protein in octocorals, we downloaded all publicly available mitochondrial genomes of octocorals (89 species) from GenBank (supplementary file S1, Supplementary Material online).

### Identification of MutS Homologs

#### Phylum Cnidaria

The protein models from the 17 cnidarian species and the four reference species were used as input for OrthoFinder v2.4.0 to identify groups of orthologous proteins (OGs) in cnidarian and reference species (using default parameters) (Emms and Kelly 2019). OGs containing MSH proteins from reference species were extracted for further analysis. BLASTP was used to identify homologs of the MSH proteins from these OGs in the Non-Redundant protein database (NR) (e-value: 1e^−5^) (Altschul et al. 1990; Camacho et al. 2009) and remove potential contamination. We retained only those proteins with a top BLASTP hit from Cnidaria. Since some of the cnidarian MSH proteins were fragments, we created a manually curated set of MSH proteins from cnidarians for the downstream phylogenetic analysis (supplementary folder S3, Supplementary Material online).

In addition, we used an HMMer-based approach to further examine instances of absence of MSH proteins in the analyzed genomes (referred to as the HMM approach). For this, we downloaded reviewed sequences of MSH1–6 from Uniprot. Using these reviewed entries and MSH proteins identified in two well-studied cnidarians, *Nematostella vectensis and Hydra vulgaris*, we built HMM profiles of each MSH protein (MSH1–6) using HMMer v3.1.2 (Eddy 2011). These profiles were then used to search for MSH proteins in the animal genomes (e-value: 1e^−5^). For each animal genome, the results of the HMMer searches were downloaded, filtered based on sequence length (proteins below 500 amino acids in length were removed), and aligned to sequences of MSH1–6 from human and yeast and MSH1–6 + mtMutS from the octocoral *Dendronephthya gigantea*, using MAFFT v7 (“–auto” option) (Katoh and Standley 2013). RAxML v8.2.11 was then used to build a phylogenetic tree for the resulting alignment with automatic selection of the substitution model and rapid bootstrapping with 100 resamples (“-m PROTGAMMAUTO -p 12345 -x 12345 -# 100”) (Stamatakis 2014). The resulting phylogenetic trees were manually inspected to identify MSH subunits.

We extracted the mtMutS gene from the 89 publicly available octocoral mitochondrial genomes. For this, we reannotated all mitochondrial genomes using MITOS2 (Bernt et al. 2013). We identified the MutS gene in all but one octocoral species, *Paragorgia* sp. USNM 1075769. The mtMutS gene from the latter species was riddled with multiple stop codons, possibly from misassembly of the mitochondrial genome. Hence, we excluded it from the final data set.

#### Other Species

OrthoFinder v2.4.0 was used to identify groups of orthologous proteins within the 57 animal and the 8 outgroup species (supplementary file S2, Supplementary Material online). OGs containing MSH proteins from reference species were extracted for analysis and the presence or absence of MSH1–6 was recorded. As described in the previous section, we used the “HMM approach” to further investigate the distribution of MSH subunits in these genomes.

### MSH1 and mtMutS Characterization

Multiple tools were used to predict the subcellular localization of the MSH1 identified in this study: DeepMito (Savojardo et al. 2020), TargetP v2.0 (Emanuelsson et al. 2007), and MitoFates (Fukasawa et al. 2015). TargetP was used using the “Nonplant” option, DeepMito was used with default parameters, and MitoFates was used with the “Metazoa” option for animal sequences and “Fungi” option for the *S. cerevisiae* sequences. For identifying protein domains, we downloaded HMM profiles of “MutS I”, “MutS II”, “MutS III”, “MutS IV”, and “MutS V” (the protein domains found in yeast MSH1). We used HMMer to identify these domains in the protein sequences of interest. Protein alignments were visualized by ESPirit v3 (Robert and Gouet 2014). Pairwise percentage identity to yeast MSH1 was calculated based on pairwise alignments of each individual MSH1 protein with yeast MSH1.

### Phylogenetic Analysis

BLASTP was used to identify homologs of the hexacoral MSH1 and octocoral mtMutS proteins from the NR database. Using the octocoral mtMutS as a query, the top ten best BLASTP hits (based on e-value) from (i) bacteria, (ii) archaea, (iii) viruses, and (iv) eukaryotes were extracted from the NR database. Using the hexacoral MSH1 as query, the top ten best BLASTP hits from the NR database for each of the four categories mentioned above were downloaded (supplementary folder S4, Supplementary Material online). The manually curated set of MutS homologs from cnidarians and the protein sequences resulting from the BLASTP searches were aligned using MAFFT v7.453 (“–auto” option). TrimAI v1.2 was used to remove the poorly aligned positions in the alignment (“automated1” option) (Capella-Gutiérrez et al. 2009). The phylogenetic tree was reconstructed using RAxML v8.2.11 with automatic selection of the substitution model and rapid bootstrapping with 1,000 resamples (“-m PROTGAMMAUTO -p 12345 -x 12345 -# 1000”). RAxML identified the LG model as the best-scoring model of substitution. The resulting phylogenetic tree was visualized in iTOL v5.7 (Letunic and Bork 2019).

BLASTP was used to identify the top ten best BLASTP hits (based on e-value) from prokaryotes, viruses, and eukaryotes, from the NR database for MSH1–6 from yeast and *Nematostella vectensis*. CD-HIT was used to cluster the sequences of the BLASTP hits and the homologs of mtMutS identified from the NR database (described above) at 80% (“-c 0.8 -n 5”). These resulting sequences, along with MutS homologs from human, yeast, *N. vectensis*, and the octocoral *Dendronephthya gigantea* (MSH1–6 and mtMutS) were aligned using MAFFT (“–auto” option). TrimAI v1.2 was used to remove the poorly aligned positions in the alignment (“automated1” option). RAxML v8.2.11 was used to construct the phylogenetic tree with automatic selection of the substitution model and rapid bootstrapping with 500 resamples (“-m PROTGAMMAUTO -p 12345 -x 12345 -# 500”).

## Supplementary Material

Supplementary data are available at *Genome Biology and Evolution* online.

## Data Availability Statement

The sequences, scripts, and other supplementary materials from this study can be found on the project repository at Open Science Framework (https://osf.io/rn9ft/): Muthye V, Lavrov D (2020) Data for Dynamic evolution of the MutS family in animals: multiple losses of MSH paralogs and gain of a viral MutS homolog in octocorals. doi : 10.17605/OSF.IO/RN9FT.

## Supplementary Material

evab191_Supplementary_Data
